# Supplementary material for: Association between biological sex and insecticide-treated net use among household members in ethnic minority and internally displaced populations in eastern Myanmar
Source: PLoS One. 2021 Jun 18;16(6):e0252896. doi: 10.1371/journal.pone.0252896 (PMC8213136; doi:10.1371/journal.pone.0252896)
Supplement: Attachment — Submitted filename: FINAL Response to Reviewers_PLOS One.docx [file pone.0252896.s001.docx]

March 27, 2021

PONE-D-20-40284
Association between biological sex and insecticide-treated net use among household members in ethnic minority and internally displaced populations in eastern Myanmar

To the Editor and Reviewers:

Thank you very much for the thorough review of our manuscript. We have carefully read and responded to each of the reviewers comments and suggestions. Our responses to each are detailed below and we have uploaded a track changes version of the edited manuscript. We do hope this helps to satisfy the reviewers concerns and we look forward to hearing back from you about this manuscript.

Sincerely,

Dr. Colleen Davison (corresponding author)

Breagh Cheng (lead author)

Naw Pue Pue Mhote (co-author)

Saw Nay Htoo (co-author)

**Editor Comments**

1. Check PLOS ONE journal style requirements

- File naming
- Check style templates

We have reviewed the PLOS One journal style requirements, including file naming and style templates and have made adjustments as needed.

2. Provide justification for reasons for not doing any post-hoc corrections for multiple comparisons

Thank you for your comment. Six comparisons were instigated in this analysis. Post-hoc corrections for multiple comparisons were not conducted due to the small number of planned comparisons and the intention for the results to inform future study. We have included this information as a note of caution in the limitations section on page 21.

3. State if we accounted for clustering by locality ie. did I consider using multilevel models

We used multilevel modelling with fixed effects with random intercept for our analysis to account for clustering across enumeration areas and ensure robust standard errors. In this study, enumeration areas consisted of strata, defined as catchment areas serviced by ethnic and community-based organizations. We have noted our approach to statistical analysis and provided clarification on enumeration areas in this study under the statistical analysis heading in the methods section on page 9.

4. Statistical analyses need a thorough review. I have noticed several errors across the tables. Examples:

- the 95% CIs are not consistent with the point estimates in Table 1 for age 50+ (did not use ITN).
- Table 2, Lives in household with sufficient ITN supply for comparison by sex of HH member, the 95% Cis overlap, but p-value is 0.03 which does not look correct.

Thank you for the comments. We have conducted a thorough statistical review and have made several changes to improve the clarity and accuracy of our statistical reporting.

First, we would like to note that the 95% confidence intervals in Table 1 and 2 presented in the Results section are provided for frequency and percentages, rather than point estimates. We have provided 95% confidence intervals in Table 3 (page 17). All associations that were statistically significant did not include the null point estimate of 1 in the 95% confidence intervals and had a corresponding p-value below 0.05.

To further clarify our statistical reporting, we have removed two columns indicating non-use of ITNs (and kept ITN use columns) in Table 2 (page 15) to improve clarity and for formatting purposes. Given that we have already presented frequency and percentages for ITN use, we believe it is redundant/unnecessary to present information on non-use.

We have updated frequency and percentages in Tables 1, 2, and 3 after identifying errors due to data entry. Due to the slightly updated sample size, we have updated the statistical analysis, p-values, and effect estimates for Tables 2 and 3. These changes are also reflected accordingly in the Abstract, Results and Discussion section. We have removed the ICC calculation at the cluster-level (and retained and updated the ICC for the household level).

**Clarify the following:**

5. How the missing values were treated in the analysis?

We agree that adding an explanation for how missing values were treated in analysis is needed to clarify interpretation of our results. Missing values were defined as responses indicating “don’t know”, “not available”, “refused,” or missing and were excluded from analysis. We have added an explanation of how missing values were treated to page 9 under the statistical analysis heading in the methods section.

6. Give more clarity on the sampling of households

We have added a section to page 5 under the data source and sampling technique heading beginning that clarifies the sampling strategy for the data used in this analysis.

7. Why local irb approval was not taken for this study?

Thank you for raising this point. To clarify, IRB approval was obtained from both the institution conducting the research and the ethics committee based in Mae Sot, Thailand overseeing access to the dataset used for the analysis. We have changed the ethics statement section on page 9 under the ethics statement section so that local ethics approval is made clear in the first sentence.

8. Who was responsible for collecting data and when exactly data was collected, give clarity around it

A new data collection heading was created in the methods section on page 6, where additional information was added to clarify the data collection process and the time periods the data were collected.

9. Clarify if the duration of data collection had any effect on the itn use or other survey measures?

Thank you for the comment, we agree this is an important point that would clarify the interpretation of our study results. We added a sentence to page 6.

10. In the discussion line 271, you suggest that ITN use was self-reported. Does it mean all HH members were asked questions on ITN use? If not, then change it accordingly.

We have removed the sentence of concern.

**Reword the following:**

11. “…multivariable analysis instead of multivariate analysis”

This change was made on page 16.

12. Rename Table 0 as Table 3?

We have checked the numbering of tables and confirm that they are properly ordered from Table 1 to Table 3. Table 1 is placed on page 10, Table 2 is placed on page 15, and Table 3 is placed on page 17.

13. Include interaction analysis result in the table

Thank you for the suggestion, we agree it would be important to report the full results of the interaction analysis. Due to formatting challenges, we were unable to include interaction results in Table 3. Instead, we have added the results of the interaction analysis as a footnote under Table 3 on page 17.

**Clarify the following**

14. A key limitation is the use of a data that may not be representing the current situation. Suggest including this as a limitation.

This was added as an additional limitation in the limitations section on page 20.

15. We note that you have indicated that data from this study are available upon request. PLOS only allows data to be available upon request if there are legal or ethical restrictions on sharing data publicly. For more information on unacceptable data access restrictions, please see link

We have taken note of PLOS’s data publishing policies and would like to confirm that the data used in this study is available upon request. Access to the dataset is governed by the local ethics committee and members of the working group that oversaw its collection and possess ownership of the data. They would field any requests and make decisions about who would be able to gain access to these data in the future. We have clarified this further in the ethics statement on page 10.

16. This is an interesting area of work.

Thank you for the comment, we appreciate your interest in our work.

17. The paper needs a strong justification on why you are using data from 2013. Given the development that has taken place in Myanmar in last few years, does this data still hold significance? Please include justification around it.

We have included additional rationale on page 19, with supporting rationale on page 21 to justify the relevance and use of data from 2013.

18. If there are ethical or legal restrictions on sharing a de-identified data set, please explain them in detail (e.g., data contain potentially sensitive information, data are owned by a third-party organization, etc.) and who has imposed them (e.g., an ethics committee). Please also provide contact information for a data access committee, ethics committee, or other institutional body to which data requests may be sent

The de-identified version of the data set used in this study is not available to the general public. While data is de-identified, it contains potentially sensitive information on ethnic communities in Myanmar, including refugees and persons who have been forced to immigrate due to conflict.

Data is collected and owned by the Health Information Systems Working Group in Mae Sot, Thailand, which is comprised of several ethnic and community-based organizations servicing areas in eastern Myanmar. Researchers interested in using this dataset must obtain written permission and approval from the local ethics committee and the Health Information Systems Working Group themselves. We work closely with HISWG and both BC and CMD spent time in Mae Sot to develop and conduct these analyses with our partners there. We have listed the contact information for the ethics committee under the ethics section and added additional clarification on page 10.

19. If there are no restrictions, please upload the minimal anonymized data set necessary to replicate your study findings as either Supporting Information files or to a stable, public repository and provide us with the relevant URLs, DOIs, or accession numbers. For a list of acceptable repositories, please see

As stated above, the anonymized data set is not shared publicly. A request can be made to HISWG, ethical approval must be obtained from the Mae Tao Clinic Ethics Committee and the Health Information Systems Working Group before access is allowed.

20. We note you have included a table to which you do not refer in the text of your manuscript. Please ensure that you refer to Table 0 in your text; if accepted, production will need this reference to link the reader to the Table.

We have reviewed the table numbering in the manuscript and confirm they are numbered accordingly from Table 1 to 3. All tables have been referenced in the text of the manuscript.

21. Please include a copy of Table 3 which you refer to in your text on page 12.

We have reviewed numbering of our Tables and their references in the report and confirm that they are appropriately numbered and referenced.

**Reviewer #1**

22. The authors explored ITN access and use among a high risk migrant population in Myanmar using data from 2013. They demonstrate differences in ITN use by age and biologic sex and that pregnant women and children under five are prioritized to use ITNs. Their findings may potentially be used inform the distribution strategies and promote use of ITNs among this vulnerable population.

Thank you for your comments.

23. The manuscript will hugely benefit from a detailed justification of their use of data from 2013, particularly given the change in malaria landscape since 2013.

- The authors note that Between 2010-2018, the number of reported malaria cases in the country declined by 76% and deaths fell by 95%. The context of the migrant population in Myanmar has also changed with the Rohingya crises and over one million people in need of humanitarian assistance.
- This manuscript would be bolstered significantly by the use of more recent data or a clear rationale for the use of such dated data, delineating how the study findings are still relevant in 2021.

We agree that this is an important point that will improve the strength of our study findings As stated above, we have included additional rationale to page 19 to justify the relevance and use of data from 2013.

- It would also be helpful to note any differences in the study population, ITN distribution channels and major factors influencing ITN access and use have changed from the time of the study to date.
- Of note, the study findings probably do not reflect the more recent shift to universal coverage of ITN ownership and use which may have occurred since 2013.

We agree that these additional details may help with interpretation of our study results in light of changes since the data were collected in 2013. We have added additional information that highlights changes in mass population movement (on page 20), additional predictors of ITN access, and use (on page 19) and ITN distribution strategies used in Myanmar (on page 4).

Universal ITN coverage has been implemented in Myanmar prior to collection of the data used in this study. The ethnic minority groups we are reporting on are historically “last mile” populations for health interventions in the country. Within the context of universal coverage, our study provides information on (in)equitable ITN access and use and also whether certain demographic sub-groups, such as pregnant women, are prioritized within the household.

24. Current thinking as evidenced by the recent literature and global research such as Malaria Indicator Surveys, Demographic and Health Surveys and Multiple Indicator Cluster Surveys now highlight population level access to ITNs. I would encourage the authors include overall population level ITN access in their findings.

- Of note, while this indicator does not allow exploration of individual level factors, it might benefit the manuscript to show how the population level ITN access and potential differences by household or regional level factors. For more information on the methodology, see [https://journals.plos.org/plosone/article?id=10.1371/journal.pone.0097496:](https://can01.safelinks.protection.outlook.com/?url=https%3A%2F%2Fjournals.plos.org%2Fplosone%2Farticle%3Fid%3D10.1371%2Fjournal.pone.0097496%3A&data=04%7C01%7Cbreagh.cheng%40queensu.ca%7C831ea49c0f91466f347808d8d29d757c%7Cd61ecb3b38b142d582c4efb2838b925c%7C1%7C0%7C637490921014401093%7CUnknown%7CTWFpbGZsb3d8eyJWIjoiMC4wLjAwMDAiLCJQIjoiV2luMzIiLCJBTiI6Ik1haWwiLCJXVCI6Mn0%3D%7C1000&sdata=qVuTP3llyDQgooBFPtr2pPrYmEQVWXzayyNYWmGitA4%3D&reserved=0)
- First, an intermediate variable of “potential ITN users” was created by multiplying the number of ITN in each household by a factor of 2.0. In order to adjust for households with more than one net for every two people, the potential ITN users were set equal to the de-facto population in that household if the potential users exceeded the number of people in the household.
- Second, the population access indicator was calculated by dividing the potential ITN users by the number of de-facto members for each household and determining the overall sample mean of that fraction.

Thank you for the suggestions. We have included a calculation of population ITN access in the results section on page 12 using the recommended resource you have provided.

25. Line 77-78. Consider revising the sentence: A key challenge to this goal is the effective use of ITNs. It reads as if the major challenge of the goal of using ITNs is the use of ITNs which is probably not what the authors intended.

We have removed this sentence and reworded the corresponding paragraph on page 4 to better communicate our intended meaning.

26. Some of the columns do not add up to 100%. I would recommend the authors make the characteristic- Other socio- demographic sub-groups- mutually exclusive by including an "other" category and revising the characteristic to "socio- demographic sub-groups".
    Also, the final characteristic- household members- should be revised in order to add up to 100%.

We agree that making the group mutually exclusive will greatly reduce any confusion on reporting of characteristics for sociodemographic subgroups. We have renamed the heading title and revised the Household Members section to add up to 100% as suggested.

27. Include the p values for each of the other socio-demographic sub-groups

After making the socio-demographic sub-groups mutually exclusive, we have included the p-values, which have been moved into the sub-headings to indicate whether there were overall significant differences in ITN use by sex, age, or socio-demographic sub-groups.

28. As noted earlier, please discuss the dated findings in light of potential changes to the study population context, ITN distribution strategies, and factors influencing ITN access and use.

As mentioned earlier, we have added additional information that highlights changes in mass population movement (on page 20), additional predictors of ITN access and use (on page 19) and ITN distribution strategies used in Myanmar (on page 4).

29. This is notably absent from the main text. Kindly include

A conclusion was added on page 21.

**Reviewer #2**

30. This was a well-written paper examining a question that is of interest currently - namely the role of gender on ITN use. Also, Myanmar is a country about which little is known on the subject of malaria prevention. This paper provides some interesting insights.

We appreciate your interest in our work.

**Reviewer #3**

31. The abstract section needs to be revised and it should be as per PLOS One style and format

We have updated the abstract based on the statistical and discussion changes.

32. In Materials and methods section, in page 6: the authors can add Measures section before the variables used and dependent variable/outcome variable sub section and independent variable/explanatory variable section (effect modifiers and confounding variables) section should be created.

We have reordered the headings and created separate sub sections for dependent and independent variables on page 7 based on your suggestion.

33. Again, it will be good if the authors can write detail description on the variables used in the analysis (like categories, definition etc.)

We have added additional clarification to the methods section for independent and dependent variables to explain how variables were categorized and defined (on page 7).

34. The current form of results and discussion section looks weak. If the additional analysis on prevalence of malaria in the region/geography can be added (for sex and geography), along with trend, pattern of malaria prevalence/incidence it will be good.

- Additionally, the association between ITN use and malaria prevalence/incidence will be very good for future policy and program enhancement and take away.

We agree this would strengthen the results and discussion of this paper. We have noted differences in malaria risk among age/sex groups (on page 4). We have also clarified our study’s findings on ITN use by highlighting patterns in malaria risk by age/sex group on page 18.

35. Without the revised results and analysis, the paper discussion section feels like half knowledge has been provided in the region. The discussion section needs to be strengthened with the revised analysis and findings. Then possibly the recommendations and conclusion can be better placed.

We hope that the added information on rationale for the study’s findings relevance, ITN distribution channels, malaria prevalence, comparison of results to malaria incidence from other data sources and time periods has strengthened the discussion.
